# Advanced immunotherapy across diseases and the role of artificial intelligence: A review

**DOI:** 10.17305/bb.2026.13199

**Published:** 2026-01-28

**Authors:** Ritika Sharma

**Affiliations:** 1 SKAN Research Trust, Bengaluru, Karnataka, India

**Keywords:** Immunotherapy, cancer immunotherapy, autoimmune disorders, infectious diseases, artificial intelligence

## Abstract

Immunotherapy, a therapeutic strategy aimed at modulating the host immune system, has undergone rapid evolution over recent decades, particularly in oncology. Advanced methodologies, including immune checkpoint inhibition, cytokine therapy, chimeric antigen receptor T-cell therapy (CAR-T), and tumor-infiltrating lymphocyte therapies, have significantly transformed cancer treatment. This review summarizes recent advancements in immunotherapy and examines its expanding applications across a range of diseases, such as autoimmune disorders, infectious diseases, transplant rejection, and allergic conditions. A structured literature search was conducted using PubMed and Google Scholar, prioritizing studies published from 2015 to 2026. The findings underscore the efficacy of monoclonal antibodies, adoptive cell therapies, cytokine modulation, and checkpoint-targeted strategies beyond oncology. However, challenges remain, including variable patient responses, immune-related adverse events, and treatment costs. This review also explores the emerging role of artificial intelligence (AI) in enhancing personalized immunotherapy through patient stratification, biomarker identification, and predictive modeling. The integration of multi-omics data with AI presents promising opportunities for improving treatment efficacy and safety, although issues related to data quality, interpretability, regulatory frameworks, and ethical considerations must be addressed. In conclusion, immunotherapy is rapidly extending beyond cancer, and AI-supported personalized approaches offer a promising pathway to safer, more effective, and broadly applicable treatments.

## Introduction

The therapeutic approach of harnessing the host’s immune system for protection against disease, through the initiation, enhancement, or suppression of immune responses, is termed immunotherapy. This field has advanced significantly since its inception, particularly regarding its efficacy in cancer treatment. Currently, immunotherapies are being employed to combat a variety of diseases [1].

This review aims to highlight the progress made in immunotherapy and focus on how these advancements are being explored to develop superior therapeutics for diverse diseases. Additionally, the intersection of artificial intelligence (AI) with immunotherapy and precision medicine will be discussed.

## Search methodology

A structured literature search strategy was employed to compile this study. Relevant studies focusing on immunotherapy, cancer immunotherapy, immunotherapy beyond cancer, AI, and the intersection of AI and immunotherapy were screened. Google Scholar and PubMed were utilized to retrieve primary literature. Keywords and Boolean operators included: “immunotherapy,” “history of immunotherapy,” “immunotherapy AND diverse diseases-cancer,” “immunotherapy NOT cancer,” “immunotherapy AND artificial intelligence,” “immunotherapy AND artificial intelligence OR predictive models-cancer,” “artificial intelligence AND healthcare,” and “explainable artificial intelligence.” No strict inclusion criteria were applied; however, preference was given to articles published between 2015 and 2026 to ensure updated context. Only articles written in English were selected, and those not published in a peer-reviewed, indexed journal were strictly excluded.

## Historical perspective

**Figure 1.**
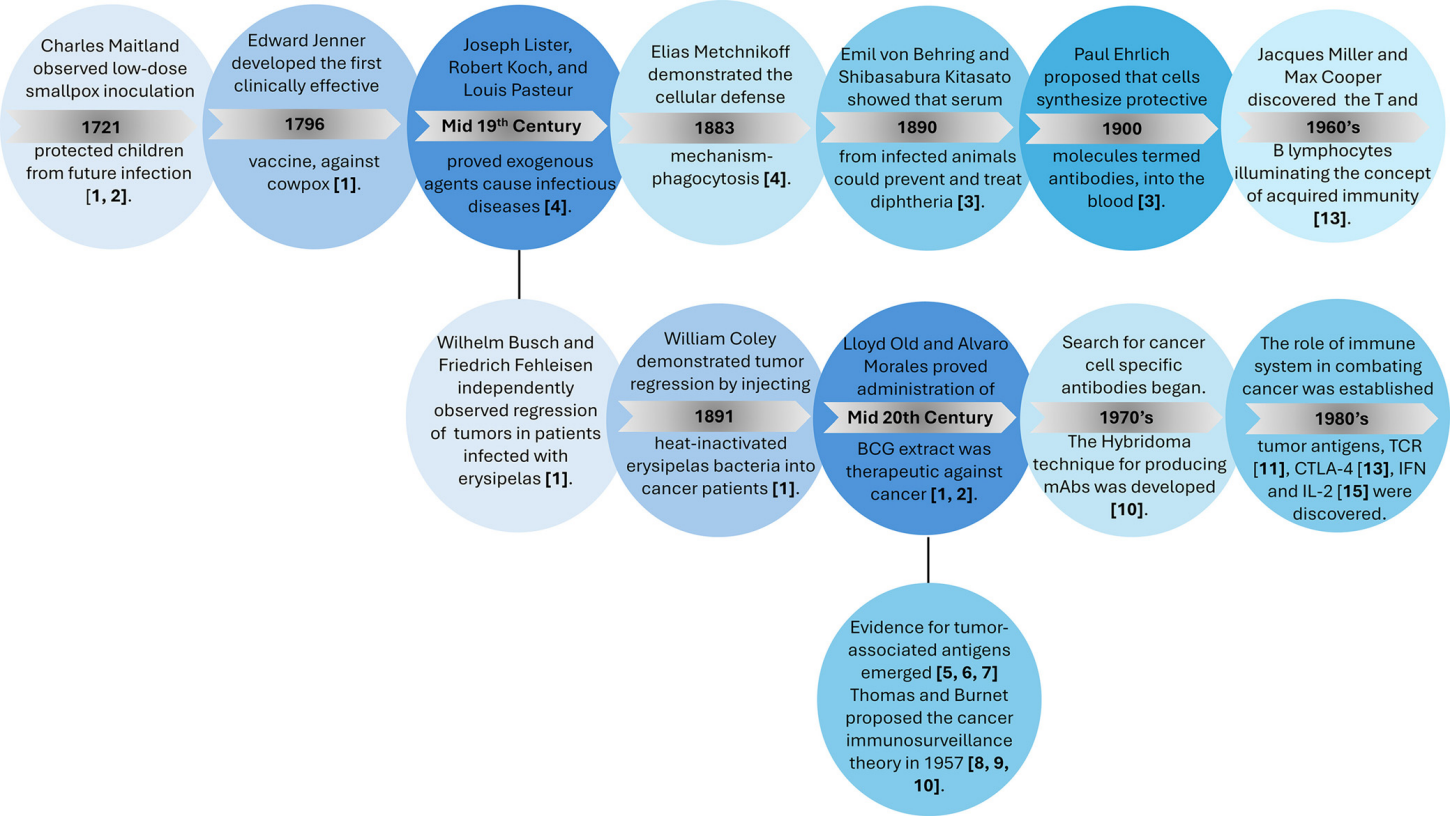
**Chronological overview of landmark discoveries that shaped modern immunology and immunotherapy.** The upper panels trace the recognition of infectious diseases and host defense mechanisms—from early variolation and Jenner’s smallpox vaccination, through the germ theory of disease, discovery of phagocytosis, antibodies and serum therapy, to the identification of T and B lymphocytes. The lower panels highlight pivotal advances in cancer immunotherapy, including observations of infection-induced tumour regression, the therapeutic use of BCG, the description of tumour-associated antigens and cancer immunosurveillance, the advent of hybridoma technology and monoclonal antibodies, and the discovery of TCR, CTLA-4 and key cytokines that enabled modern immune-based therapies. Dates indicate the approximate time of each discovery; bracketed numbers correspond to the primary references cited in the Historical Perspective section. *Figure created by the authors from cited sources.* Abbreviations: BCG: Bacillus Calmette–Guérin; TCR: T-cell receptor; CTLA-4: Cytotoxic T-lymphocyte-associated protein 4.

The origins of immunotherapy date back to 1721 when Charles Maitland intentionally infected children with low doses of smallpox to prevent them from contracting the disease [1, 2]. Research in the mid-19th century highlighted infectious diseases, yet the concept of a host defense mechanism against such invaders was not established at that time. The later discovery of phagocytic cells and antibodies confirmed the existence of an inherent host defense mechanism [3], leading to the emergence of immunology as a distinct discipline [4] (Figure 1).

The dawn of cancer immunotherapy occurred in the late 19th century when Busch, Fehleisen, and Coley independently observed tumor regression in patients following infection with erysipelas. However, their findings did not receive significant attention at the time [1, 2]. Interest in cancer immunotherapy revived in the mid-20th century with the presentation of evidence for tumor-associated antigens [5, 6, 7]. This was followed by the proposal of the immunological surveillance theory, which posited that lymphocytes play a crucial role in identifying and eliminating malignant cells [8, 9, 10]. The search for antibodies with binding specificity to malignant cells also commenced. In 1975, hybridoma technology revolutionized therapeutic antibody research, ultimately leading to the development of rituximab, the first Food and Drug Administration (FDA) approved monoclonal antibody for cancer treatment (non-Hodgkin’s lymphoma) [11]. A remarkable breakthrough in cancer immunotherapy followed with the discovery of the T-cell antigen receptor [12] and cytotoxic T-lymphocyte-associated protein-4 (CTLA-4) [13]. These findings paved the way for further research and resolved the debate regarding the feasibility of utilizing the immune system to combat cancer [14]. Additionally, the discovery of interferon (IFN) and interleukin-2 (IL-2) led to the development of cytokine-based immunotherapies [15]. In 1986, interferon alpha-2 (IFN-α2) was approved by the FDA for treating hairy cell leukemia, marking the first approval of an immunotherapeutic drug [2].

The 20th century also witnessed advances in allergen immunotherapy. In 1911, Leonard Noon identified grass pollen as the cause of hay fever and noted the protective effects of its crude extract preparations. Drawing insights from this work, William Frankland conducted the first randomized, double-blind, placebo-controlled immunotherapy trial, demonstrating the therapeutic efficacy of subcutaneous grass pollen injection therapy. Subsequently, allergy shots became the standard treatment for allergies [16].

## Basic to advanced immunotherapy

**Figure 2.**
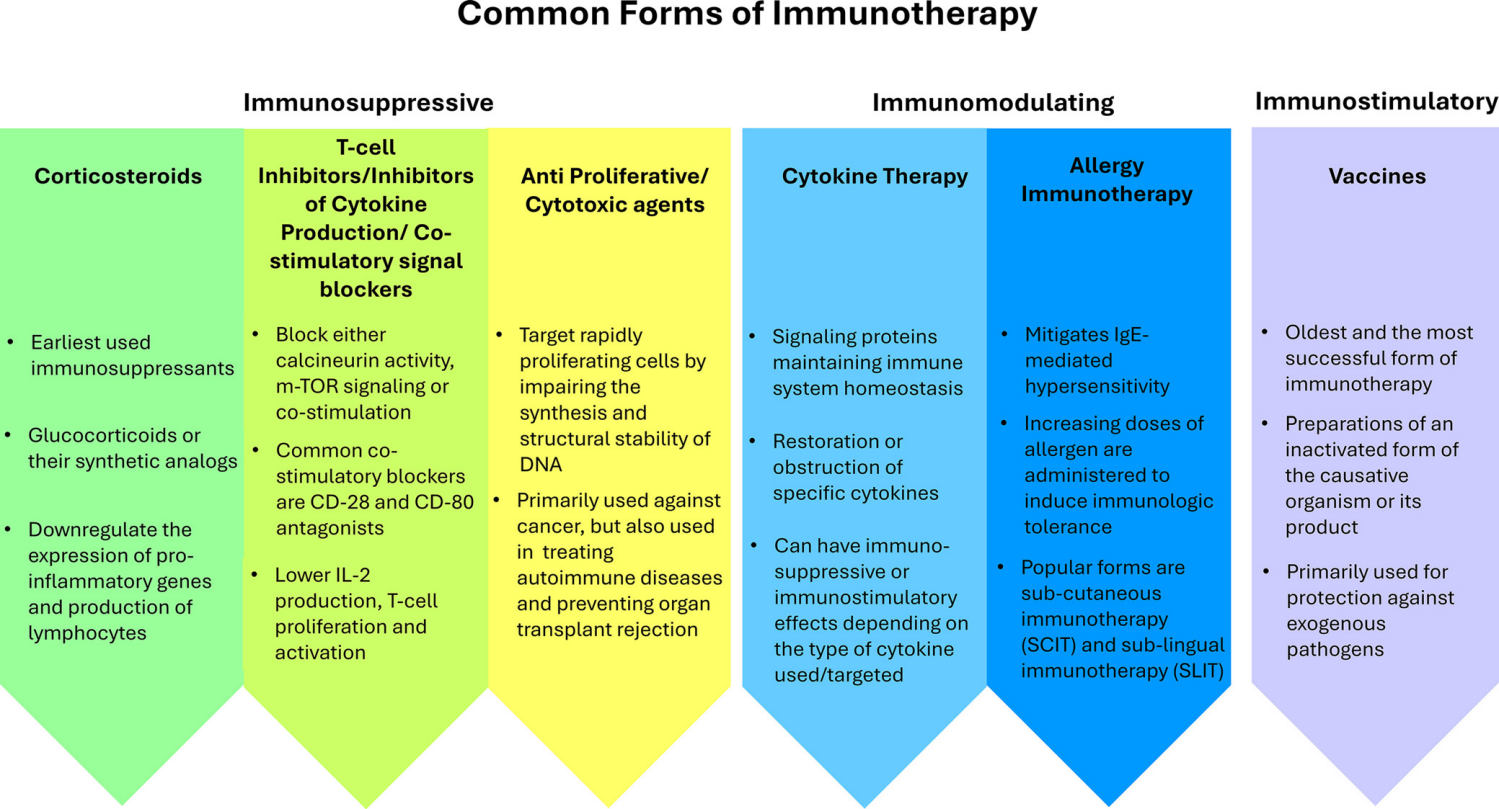
**Schematic overview of common forms of basic immunotherapy, grouped according to their predominant effect on the immune system.** Immunosuppressive approaches include corticosteroids, T-cell inhibitors that block calcineurin, mTOR signalling or co-stimulation (CD28/CD80 antagonists), and anti-proliferative/cytotoxic agents used in cancer, autoimmune disease and prevention of allograft rejection. Immunomodulating approaches comprise cytokine therapy, which restores or blocks specific signalling proteins to re-establish immune homeostasis, and allergen immunotherapy, which gradually increases allergen exposure (e.g. via SCIT or SLIT) to induce long-term tolerance and reduce IgE-mediated hypersensitivity. Immunostimulatory approaches are exemplified by vaccines, in which inactivated or attenuated pathogens or their components are administered to elicit durable protective immunity against subsequent infections. *Figure created by the authors from cited sources.* Abbreviations: IgE: Immunoglobulin E; SCIT: Subcutaneous immunotherapy; SLIT: Sublingual immunotherapy; mTOR: Mechanistic target of rapamycin.

Immunotherapy can take various forms depending on the disease condition (Figure 2). Immunotherapeutics are primarily categorized as immunostimulants (e.g., vaccines) and immunosuppressants (e.g., corticosteroids). Cytokine therapy and allergy immunotherapy are classified as immunomodulating approaches.

Cancer immunotherapy is the most prominent form of immunotherapy and has been at the forefront of advancements in this field. Most clinically approved cancer immunotherapies utilize T cell functions and can be broadly categorized into two types: modulators of endogenous T cell responses and cellular therapies [17]. Immune checkpoint inhibitor (ICI) therapy, cytokine therapy, cancer vaccines, and oncolytic virus therapy fall under the category of modulators of endogenous T cell responses, while chimeric antigen receptor T-cell (CAR-T) therapy and tumor-infiltrating lymphocytes (TIL) therapy represent the principal forms of cellular therapies [18].

### Checkpoint inhibitor therapies

Cancer cells exploit cell-surface immunoregulatory proteins known as checkpoint molecules to evade the immune system; these proteins inhibit T-cell function. CTLA-4 and programmed cell death-1 (PD-1) were the first immune checkpoint molecules identified, leading to the development of therapeutic monoclonal antibodies (mAbs) known as checkpoint inhibitors [19]. mAbs such as anti-CTLA-4, anti-PD-1, or anti-PD-L1 impede the activity of these immune checkpoints. In 2011, Ipilimumab, an anti-CTLA-4 mAb, became the first FDA-approved ICI therapeutic for advanced melanoma. Since then, over six checkpoint inhibitors have received FDA approval as neoadjuvant and/or adjuvant therapies for various malignancies [20]. Despite their revolutionary impact, checkpoint inhibitor therapy has limitations that affect its broader applicability [21, 22].

### Cytokine therapy

Cytokines serve as molecular coordinators among immune cells, triggering self-limited, highly specific immune responses. There are seven different types of cytokine receptors, and targeting cytokines or their receptors has been crucial in developing anti-cancer therapeutics [1, 23]. The FDA has approved several cytokines for treating various malignancies, including high-dose IL-2 for metastatic melanoma and IFN-α for renal cell carcinoma [24], as well as IFN-α2a and IFN-α2b for patients with hairy cell leukemia [1, 21]. Recently, the combination of Bacillus Calmette-Guérin (BCG) with an IL-15 super agonist termed N-803 received approval for treating non-muscle invasive bladder cancer [23, 25].

### Cancer vaccines

Cancer vaccines consist of formulations of whole tumor lysates (from either the patient’s own tumor or another source), tumor-specific antigens, and viral vectors, among others. When administered with adjuvants, these vaccines can elicit anti-tumor immune responses by activating T cells [21]. In 2010, Provenge (Sipuleucel-T) became the first FDA-approved cancer vaccine for treating prostate cancer [26]. Intravesical BCG for non-muscle invasive bladder cancer is another FDA-approved immunotherapy [27]. Preventative vaccines targeting cancers caused by human papillomavirus (HPV) and hepatitis B virus (HBV) infections are also in clinical use [28]. Numerous promising vaccine candidates are undergoing clinical evaluation at various stages [29]. Therapeutic cancer vaccines are generally safe and do not cause significant side effects; however, outcomes may vary considerably among individuals [21].

### Oncolytic virus therapy

Oncolytic virus therapy represents an innovative immunotherapeutic approach that utilizes genetically engineered viruses to target and destroy cancer cells. When these genetically altered viruses are administered into tumors, they induce lysis of cancer cells, resulting in the release of tumor antigens. These antigens can activate immune cells, which subsequently target other cancer cells expressing those antigens. This strategy capitalizes on the fact that cancer cells are more susceptible to viral infections compared to normal cells [1]. Various viruses, including adenovirus, herpes simplex virus 1, measles virus, and reovirus, have been explored as agents of oncolytic virus therapy [30]. The first therapeutic oncolytic viral therapy to gain FDA approval was Talimogene laherparepvec (Imlygic or T-Vec), aimed at treating melanoma [1]. Several other candidates are currently undergoing clinical trials for various malignancies [1, 30].

### TIL therapy

TILs refer to the lymphocytic cell populations that invade tumor tissue. TIL immunotherapy involves isolating TILs from tumors, followed by their cytokine-mediated activation and expansion in culture, and ultimately re-administration into the patient. This therapy was first tested for melanoma treatment in the 1980s. In recent decades, the technique has been refined and adapted for treating cervical cancer and other solid tumors. In the case of melanoma, optimal response rates have increased to 50%–75% [21, 31]. This heightened efficacy is attributed to patient pre-conditioning and the depletion of lymphoid tissues [21]. Lymphodepletion reduces the number of regulatory T cells (Tregs) that suppress immune responses and other endogenous lymphocytes that can compete with the transferred TILs [32]. Despite its promise, TIL therapy faces challenges in standardization, which limits its broader applicability [21].

### CAR-T therapy

A cutting-edge immunotherapy involves the genetic modification of patient-derived T lymphocytes *in vitro* to express a chimeric antigen receptor (CAR) on their surface. Following this modification, the cells are expanded and reintroduced into the patient. A CAR consists of an extracellular, transmembrane, and intracellular domain. The extracellular domain binds to a specific antigen on cancer cells, while the transmembrane and intracellular domains activate T cells, leading to the destruction of targeted cells [21]. In 2017, the FDA approved Tisagenlecleucel (Kymriah) as the first CAR-T therapy for the treatment of B-cell leukemia. Since then, five additional CAR-T cell therapies have received regulatory approval from the FDA for various malignancies [33]. The design of CARs has evolved to enhance activation, proliferation, and survival potential. Although later generations of CARs provide superior anti-tumor effects, they are also associated with various side effects. Consequently, the clinical landscape of this therapy is currently dominated by second-generation CAR-T cells, which exhibit intermediate efficacy [34].

## Immunotherapy beyond cancer

The surge in success of cancer immunotherapy has led to a deeper understanding of immune homeostasis. The techniques developed have equipped researchers with tools to create novel and effective therapeutics for other diseases.

### Autoimmune disorders

Traditionally, immunosuppressants are preferred for treating autoimmune diseases. While these drugs can provide long-lasting remission for some conditions, their effectiveness often diminishes over time [35]. Therefore, there is a pressing need for innovative strategies to modulate the immune system for treating various autoimmune diseases. Technological advancements in immunotherapy, including checkpoint inhibitor therapy, anti-cytokine therapy, and anti-T cell therapy, have shown promising results in managing several autoimmune conditions [1].

Anti-PD-1 and anti-CTLA-4 antibodies have been developed for the treatment of autoimmune disorders. Abatacept is an mAb prescribed for specific subtypes of arthritis; it mimics the action of native CTLA-4 by interacting with co-stimulatory ligands CD80 and CD86, thereby impeding T-cell activation and inhibiting the immune response [36]. Abatacept is currently being evaluated for safety and efficacy against other autoimmune diseases [1, 17, 37]. Research studies have indicated that Abatacept has a disease-modifying effect on type 1 diabetes mellitus (T1DM) in individuals recently diagnosed with the condition [38, 39]. Additionally, Abatacept is being investigated for its safety and efficacy against systemic lupus erythematosus (SLE) and multiple sclerosis (MS). However, further research is needed to establish its therapeutic potential for these diseases [1]. Belatacept, a successor to Abatacept with superior affinity for B7 ligands, is also undergoing clinical evaluation for its therapeutic efficacy against SLE, MS, and T1DM [1, 40]. Furthermore, Peresolimab, a mAb designed as a PD-1 agonist, has shown promising results in managing rheumatoid arthritis (RA) in a phase 2a clinical trial [41].

The administration of specific cytokines or cytokine antagonists, whether alone or in conjunction with various immunosuppressants, has demonstrated favorable outcomes in managing a range of autoimmune conditions [1]. The choice of cytokine and therapeutic approach is tailored to the pathological profile of different autoimmune disorders. The principal cytokine or cytokine-directed therapies currently in use or under investigation for treating autoimmune diseases are summarized in Table 1.

**Table 1:** Cytokine therapy and cytokine-directed therapy for autoimmune diseases

**S. No.**	**Cytokine therapy/targeted cytokine**	**Autoimmune condition**	**Mechanism of action**	**Clinical status**	**References**
1	Low-dose IL-2 therapy	GvHD, SLE, T1DM	Confers Tregs with a competitive advantage leading to high Treg:Teff ratios.	Under investigation	[17, 42, 43]
2	IFN-β therapy	MS	Upregulation of Th2 anti-inflammatory response while dampening the pro-inflammatory Th1/Th17 response	Approved	[44]
3	IFN inhibition (Anifrolumab)	SLE	Complete blockade of type I IFN pathway leading to reduced inflammatory damage	Approved	[45, 46]
4	TNF-α inhibition	Psoriasis and different forms of arthritis	Blocking the pro-inflammatory action of TNF-α	Approved	[47]
5	IL-1 inhibition (Anakinra, Canakinumab)	RA and Juvenile idiopathic arthritis (JIA)	Binds to IL-1 receptor thus inhibiting the activity of inflammatory IL-1α and IL-1β cytokines	Approved	[47]
6	IL-6 inhibition (Tocilizumab, Sarilumab)	RA, JIA, SLE	Binds to IL-6 receptor/IL-6 thus inhibiting the inflammatory action of this cytokine	Approved (RA, JIA); under investigation (Tocilizumab for SLE)	[1, 47, 48]
7	IL-17 inhibition (Ixekizumab, Secukinumab, Brodalumab)	Psoriasis, Psoriatic arthritis, ankylosing spondylitis, SLE	Binds to IL-17 receptor/IL-17 thus inhibiting the inflammatory action of this cytokine	Approved (Psoriasis, Psoriatic arthritis, ankylosing spondylitis); under investigation (Secukinumab for SLE)	[47]
8	IL-23 inhibition (Ustekinumab, Guselkumab, Risankizumab, Tildrakizumab)	Psoriasis	Binds to IL-23 leading to disruption of its inflammatory signaling pathway	Approved	[47]

Abbreviations: GvHD: Graft-versus-host disease; SLE: Systemic lupus erythematosus; T1DM: Type 1 diabetes mellitus; Treg: Regulatory T cell; Teff: Effector T cell; IFN: Interferon; IFN-β: Interferon-beta; MS: Multiple sclerosis; Th1: T helper 1 cell; Th2: T helper 2 cell; Th17: T helper 17 cell; TNF-α: Tumor necrosis factor-alpha; RA: Rheumatoid arthritis; JIA: Juvenile idiopathic arthritis.

Targeted elimination of harmful B cells using mAbs [17, 49], and the application of CAR-Tregs that express high-affinity T cell receptors (TCRs) to recognize antigens responsible for triggering autoimmune responses are being tested for their therapeutic efficacy against autoimmune diseases [17, 50, 51]. Rituximab, an mAb that binds to the CD20 cell surface protein on B lymphocytes, mediates cell death through antibody-dependent cell-mediated cytotoxicity (ADCC) and/or complement-mediated cytotoxicity. It is utilized in the treatment of conditions such as RA, SLE, and MS [52]. Belimumab, another mAb, inhibits the interaction between soluble B-lymphocyte stimulator (BLyS) and the B cell receptor, obstructing the activation and survival of autoreactive B cells. Belimumab is used to treat SLE [53].

Adoptive Treg cell transfer therapy leverages the immunosuppressive role of Tregs to provide beneficial effects against autoimmune disorders. This involves the isolation of Tregs from patients, their *in vitro* expansion, and subsequent autologous transplantation back into the patient. The expansion of Tregs may involve transducing them with an appropriate auto-antigen-specific, high-affinity TCR or a CAR. These engineered, antigen-specific Tregs can deliver localized immunosuppressive effects upon reintroduction into the host [54]. This immunotherapeutic approach has yielded promising results in several preclinical studies against autoimmune diseases such as MS [55, 56], SLE [57, 58], and T1DM [59, 60, 61]. Currently, approximately 54 clinical trials are assessing the therapeutic efficacy of adoptive Treg cell transfer for various conditions associated with dysregulated immune responses [54].

### Transplant rejection

Immunosuppressive drugs have long been employed to prevent graft rejection. However, the use of these immunosuppressants is associated with significant side effects. To mitigate these adverse effects and address the low-grade immune responses that can lead to delayed allograft loss, there is a pressing need for the development of novel therapeutics. One promising approach involves targeting immune checkpoint pathways that include cell surface co-stimulatory molecules. Various co-stimulatory signaling molecules, such as CTLA-4, CD40, ICOS, OX40, T-cell immunoglobulin and mucin domain (TIM) family members, and lymphocyte function-associated antigen 1 (LFA-1), have been evaluated for their efficacy in preventing allograft rejection in preclinical studies. Several candidates are currently undergoing clinical testing [62, 63, 64]. In 2011, Belatacept (CTLA-4-Ig) received FDA approval for use as an immunosuppressant in adult kidney transplant patients [40].

### Infectious diseases

Monoclonal antibody-based therapies, checkpoint inhibition, manipulation of cytokine levels, and T-cell-based therapies are being explored as alternatives to conventional medications for infectious diseases [63].

**Figure 3.**
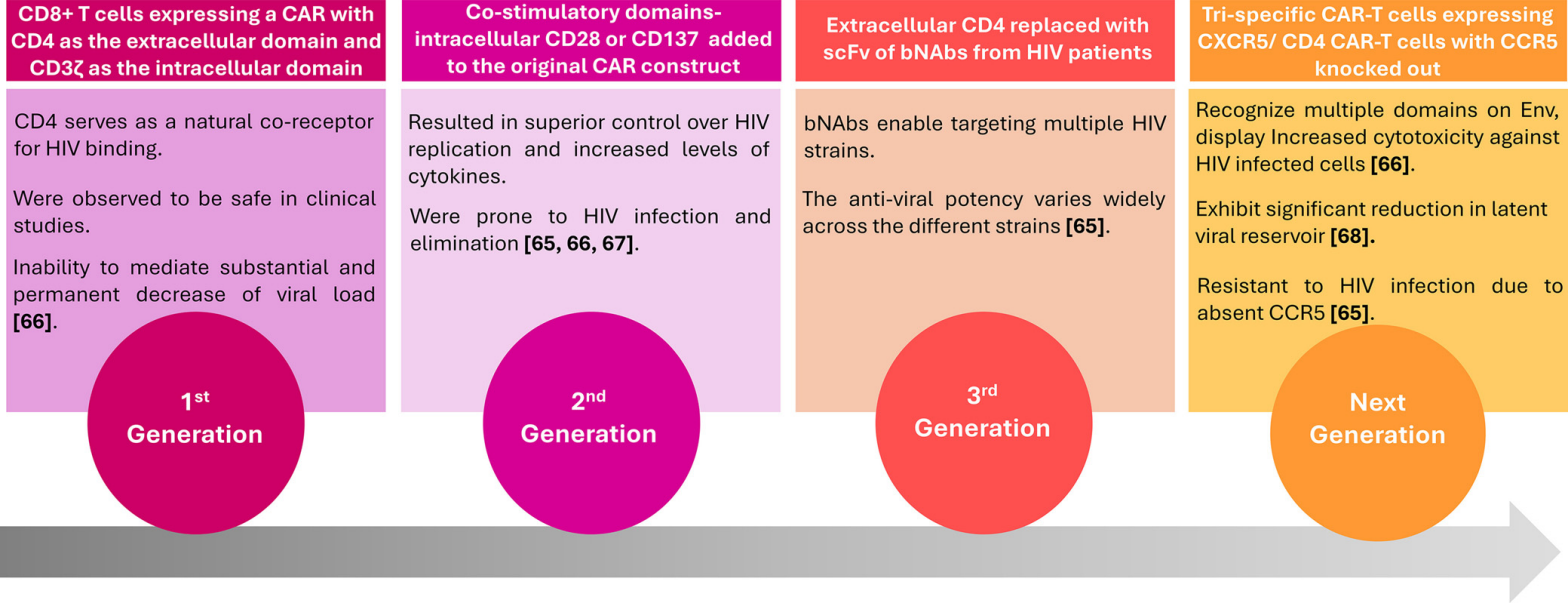
**Evolution of anti-HIV CAR-T cell designs and their clinical development status.** First-generation products comprised CD8^+^. T cells expressing a CD4-based CAR, in which the CD4 extracellular domain was linked to an intracellular signalling module derived from the native T-cell receptor complex. These constructs were safe in early trials but failed to induce substantial, durable reductions in viral load. Second-generation CAR-T cells incorporated an additional intracellular co-stimulatory domain (CD28 or CD137) into this backbone, resulting in superior control of HIV replication and increased cytokine secretion, but the cells remained susceptible to HIV infection and elimination. Third-generation constructs replaced extracellular CD4 with single-chain variable fragments from bNAbs, enabling recognition of multiple HIV strains, although antiviral potency varied between specific bNAbs. “Next-generation” strategies, currently in pre-clinical and early clinical evaluation, include trispecific CAR-T cells and CXCR5^+^ CAR-T cells with CCR5 knocked out, designed to recognise multiple epitopes on Env, home to lymphoid HIV reservoirs, enhance cytotoxicity against infected cells and resist de novo HIV infection. *Figure created by the authors from cited sources.* Abbreviations: CAR-T: Chimeric antigen receptor T cell; HIV: Human immunodeficiency virus; bNAb: Broadly neutralizing antibody; scFv: Single-chain variable fragment; Env: Viral envelope glycoprotein; CXCR5: C-X-C chemokine receptor type 5; CCR5: C-C chemokine receptor type 5.

Infectious viruses such as Human Immunodeficiency Virus (HIV), HBV, Hepatitis C Virus (HCV), and Epstein-Barr virus are being targeted using CAR-T therapy. Research on anti-HIV CAR-T cell therapy has progressed steadily since its inception in the 1990s, with three generations of anti-HIV CAR-T cells undergoing safety and efficacy evaluations in clinical trials [65, 66, 67, 68]. The next generation of anti-HIV CAR-T cells has demonstrated promising results in animal model studies [68] and has recently entered clinical trials (NCT03240328, NCT03617198). Figure 3 illustrates the key features of the different generations of anti-HIV CAR-T cells. In contrast, the development of CAR-T therapy for other viral and fungal pathogens remains in the preclinical testing stages [66].

Compared to the advancements in cancer and autoimmune disease therapies, the development of therapeutic antibodies against infectious diseases has progressed more slowly [69]. However, the COVID-19 pandemic has spurred remarkable achievements in the development, deployment, and approval of monoclonal antibodies against the SARS-CoV-2 virus [70]. Many neutralizing mAbs targeting SARS-CoV-2 were developed from memory B cells obtained from infected or recovered patients. These engineered mAbs target the spike protein of the virus, which facilitates its entry into host cells by binding to the angiotensin-converting enzyme-2 (ACE2) receptor [69]. Several of these mAbs received emergency use authorization (EUA) and played a crucial role in treating COVID-19. However, the emergence of new COVID-19 variants has diminished their efficacy. Laboratory evaluations revealed that some variants could evade neutralization by mAbs. Consequently, the FDA revoked the EUA and imposed restrictions on the use of several existing anti-SARS-CoV-2 mAbs and/or their combinations [71].

Previously, the FDA has approved mAb therapy for Ebola virus disease (EVD) [72] and HIV-1 [73]. The mAbs Nirsevimab and Palivizumab are used prophylactically against Respiratory Syncytial Virus (RSV) [74]. Additionally, several candidate antibodies against EVD, HIV, Influenza, HCV, HBV, Zika, and Dengue are currently under investigation for their safety and efficacy [65, 69, 75, 76]. Most of these antiviral antibodies are receptor-targeted and disrupt the binding and entry of viruses into host cells. Recently, broadly neutralizing antibodies (bNAbs) have garnered significant attention due to their enhanced antiviral potency against genetically diverse strains of HIV and influenza.

The FDA has approved mAbs for the treatment of bacterial infections, as shown in Table 2. Several new candidates have recently entered clinical trials, while others remain in pre-clinical development [77, 78]. The primary targets of these anti-bacterial mAbs include neutralizing toxins, membrane proteins, surface glycans or glycoconjugates, and biofilm components.

**Table 2:** Monoclonal antibodies developed as therapeutics for infectious diseases

**S. No.**	**Pathogen**	**mAb**	**Regulatory status**	**Reference**
1	**Virus**			
	SARS-CoV-2	Bamlanivimab, Bamlanivimab and Etesevimab, Casirivimab and Imdevimab, Sotrovimab	Received EUA which was later revoked by FDA	[70]
	Ebola virus	Ansuvimab (mAb114), Inmazeb	FDA-approved	[72]
	RSV	Nirsevimab, Palivizumab	FDA-approved	[74]
	HIV-1	Ibalizumab	FDA-approved	[73]
2	**Bacteria**			
	*Bacillus anthracis*	Raxibacumab, Obiltoxaximab	FDA approved	[79]
	*Clostridium difficile*	Obiltoxaximab	FDA approved	[80]

Abbreviations: MAb: Monoclonal antibody; EUA: Emergency use authorization; FDA: Food and Drug Administration; RSV: Respiratory syncytial virus; HIV-1: Human immunodeficiency virus type 1.

Additionally, mAb therapy is being assessed for its efficacy against parasitic infections, including malaria, trypanosomiasis, schistosomiasis, and leishmaniasis [81]. Multiple candidate mAbs for malaria treatment are currently under safety and efficacy review. For instance, CIS43LS, an mAb targeting *Plasmodium falciparum*, has produced promising results in phase I clinical trials [82, 83]. Another mAb, TB31F, binds to the *P. falciparum* gamete surface protein Pfs48/45, thereby impeding parasite progression and transmission. TB31F has been evaluated in a phase I clinical trial and has demonstrated safety and efficacy as a transmission-blocking mAb against *P. falciparum* (NCT04238689) [84].

Checkpoint molecules play a critical role in maintaining self-tolerance in healthy individuals, but their expression often becomes dysregulated in diseased states. Upregulation of immune checkpoint molecules leads to T cell exhaustion, which is evident in chronic infectious diseases such as HIV, malaria, hepatitis, and tuberculosis. This has prompted interest in the potential of checkpoint inhibitor therapy as a protective measure against these infections [85].

Several pre-clinical studies indicate that inhibition of checkpoint signaling enhances T cell responses against HIV [18, 86, 87]. Recently, clinical studies have supported this finding through the use of anti-cancer mAbs to mediate immune checkpoint blockade [85, 88]. A phase I trial (NCT02028403) involving HIV patients on suppressive antiretroviral therapy (ART) investigated the effects of the anti-PD-L1 antibody BMS-936559. The study found that HIV-specific CD8 T cell responses were boosted in subjects receiving the treatment, with no severe immune-related adverse events (irAEs) reported. However, BMS-936559 did not affect viral load, which may be attributed to the single, low-dose administration employed in this study [89]. Further evaluations with an optimized dosing regimen are necessary to clarify BMS-936559’s therapeutic role. Additionally, the PD-1 receptor-blocking mAb Budigalimab exhibited protective effects against HIV in recently completed clinical trials (NCT04223804, NCT04799353), demonstrating efficacy in delaying HIV rebound in participants with interrupted ART, although detailed results from these trials have yet to be published.

Checkpoint blockade has also been shown to enhance CD8 and CD4 T cell responses against HBV [85]. In separate clinical studies, the PD-1-targeting mAb Nivolumab was effective in reducing HCV and HBV loads in infected individuals [90, 91]. A recent clinical trial involving dual checkpoint blockade of PD-1 (Nivolumab) and CTLA-4 (Ipilimumab) in patients with advanced hepatocellular carcinoma (with or without hepatitis B or C) found no significant differences in overall survival among patients. However, the incidence of adverse events was higher than that associated with nivolumab monotherapy [92]. Another clinical trial examining Ipilimumab treatment in advanced melanoma patients positive for HBV and/or HCV has recently concluded, and results are pending (NCT02402699). Overall, current evidence suggests that Nivolumab is safe in the context of chronic HBV/HCV infection; however, more clinical studies are needed to validate the efficacy of immune checkpoint blockers like Nivolumab in inducing HBV/HCV remission.

The examination of immune checkpoint blockade therapy against tuberculosis has yielded mixed results, depending on the targeted checkpoint molecule. *Mycobacterium tuberculosis*-infected PD-1 knockout mice are significantly more susceptible to developing elevated mycobacterial loads and overall mortality [93, 94]. Notably, cancer patients undergoing anti-PD-1/PD-L1 blockade immunotherapy have developed atypical *M. tuberculosis* infections [93, 95]. In contrast, animal studies where T-cell immunoglobulin and mucin domain-containing protein 3 (TIM3) and lymphocyte activation gene 3 (LAG3) checkpoint functions were blocked have shown different outcomes. Blocking TIM3 in mice with chronic *M. tuberculosis* infection enhanced T cell function and significantly controlled bacterial growth [96]. Similarly, silencing *LAG3* expression in a co-culture model of CD4 T cells and differentiated macaque macrophages infected with *M. tuberculosis* triggered T cell activation and mitigated suppressive activity from regulatory T cells [97]. This disparity likely arises from the host’s baseline immune status, as evidenced by pre-clinical research and clinical studies involving cancer patients treated with ICIs [98]. In severely immunocompromised hosts, immune checkpoint inhibition appears effective in countering mycobacterial infections, whereas its application in immunocompetent hosts can induce a hyperinflammatory state and worsen control of bacterial levels [99]. These findings underscore the need for a personalized immunotherapy approach against tuberculosis infection.

Checkpoint blockade has also been explored as a potential therapeutic strategy against protist infections, though primarily in animal models. Mice infected with *Leishmania amazonensis* and treated with anti-PD-1 and anti-PD-L1 mAbs exhibited significantly lower parasite levels; however, blockade of programmed death-ligand 2 (PD-L2) did not yield similar results [100]. This discrepancy likely stems from the differing mechanisms through which PD-L1 and PD-L2 regulate immune responses during *Leishmania sp.* infections [101]. Given that leishmaniasis is a chronic infection with numerous immunoregulatory features shared with cancer, it is suggested that a “one-size-fits-all” approach may not be effective. Instead, specific combinations of ICIs may be necessary to achieve optimal responses, paralleling the need for nuanced modulation of various checkpoint inhibitory pathways to enhance outcomes against leishmaniasis [102].

The potential application of ICI therapy against malaria has been evaluated in several animal model studies; however, the evidence for efficacy remains insufficient to outweigh safety concerns. For instance, Butler et al. observed that mAb-mediated dual checkpoint blockade of PD-L1 and LAG3 increased the clearance of *Plasmodium yoelii* via enhanced CD4 T cell function and humoral immune response in C57BL/6 mice [103]. Additionally, Hou et al. noted that lymphocyte activity was restored upon blocking TIM3 signaling in cultured peripheral blood mononuclear cells (PBMCs) isolated from patients infected with *P. falciparum*, and they observed increased clearance of *P. berghei* in infected C57BL/6 mice [104]. Conversely, blockade of PD-L1/CTLA-4 checkpoint pathways in BALB/c mice had no effect on parasitemia and led to heightened T cell activation and interferon gamma (IFN-γ) levels, making the mice vulnerable to cerebral malaria [105]. Future investigations are essential to determine whether these varied findings arise from differences in the targeted checkpoint pathway, the animal model utilized, and/or species-level variations.

### Allergies

In recent decades, the prevalence of allergic diseases has risen significantly, influenced by changes in the environment and socio-economic factors [106]. This trend poses a substantial public health burden and necessitates the development of novel therapeutic approaches. Advances in immunotherapy are being explored for their safety and efficacy in this context [107].

Allergic reactions result from a predominant T helper type 2 (Th2) immune response, which arises from an imbalance among T helper type 1 (Th1), Th2, and T helper type 17 (Th17) immune functions. Th2 immune responses are driven by IL-4 and IL-13 cytokines, making them attractive targets for therapeutic intervention [108]. Consequently, antagonists of these cytokines, such as the synthetic peptide Pitrakinra, have been developed and evaluated for efficacy. However, Pitrakinra demonstrated only limited efficacy in Phase 2b clinical evaluations for patients with allergic asthma and did not advance to further stages of drug development [109]. Focus has since shifted to the development of alternative antagonists of IL-4/IL-13, including mAbs and CAR-T cell therapies [108].

mAb therapy for alleviating allergic reactions involves disrupting cytokine signaling or targeting soluble or membrane-bound IgE, the key mediators of allergic responses [110]. Several mAbs are currently available for managing allergic reactions [107].

Omalizumab is an FDA-approved humanized anti-IgE mAb used to treat moderate to severe allergic asthma. It has also shown positive effects against other conditions, including seasonal allergic rhinitis and chronic urticaria [110]. Recently, it became the first FDA-approved medication for treating IgE-mediated food allergies [111]. Ligelizumab is another IgE-directed mAb that has proven more effective than Omalizumab in managing symptoms associated with asthma and spontaneous urticaria [112]. However, phase III evaluations (NCT03580356) of Ligelizumab for the treatment of moderate-to-severe chronic spontaneous urticaria indicated that it was less effective than Omalizumab in managing the disease [113]. UB-221 is another IgE-directed mAb candidate that has shown promising results in alleviating symptoms related to chronic spontaneous urticaria [114].

Dupilumab is an interleukin-4 receptor alpha (IL-4Rα)-directed mAb that disrupts IL-4/IL-13 signaling. It has been approved by the FDA for the treatment of moderate to severe atopic dermatitis in adults and as an adjunct therapy for asthmatic patients aged six years and older [115, 116]. Moreover, Dupilumab has yielded promising results in treating chronic rhinosinusitis with nasal polyposis and allergic rhinitis [110]. Tralokinumab is the first FDA-authorized IL-13-directed mAb used for treating atopic dermatitis in adults [117].

In recent years, CAR-T therapy has been investigated in several preclinical studies for the management of allergic asthma by targeting dysregulated Tregs. This mechanism contributes to the predominant Th2 immune responses that drive allergic diseases. In murine models, directing Tregs toward inflamed airways has demonstrated efficacy in managing allergic asthma [118]. T Cells Redirected for Universal Cytokine-mediated Killing (TRUCKs) represent the fourth generation of CAR-T cells, which secrete specific cytokines. By utilizing asthma-specific biomarkers, TRUCKs can be directed to inflammatory sites, where they secrete cytokines such as IL-12 to promote Th1 cell proliferation while suppressing Th2 immune responses [119]. Additionally, targeting immunoglobulin E (IgE) producing cells has been explored as a strategy to achieve long-term suppression of IgE levels, potentially improving treatment outcomes for patients with severe allergic diseases. The transmembrane form of IgE (mIgE), expressed by all IgE producing cells, serves as a suitable target for recognition. Recently, Ward et al. developed CARs expressing the extracellular domain of FcɛRIα (a high-affinity IgE receptor) for mIgE recognition. These CAR-T cells specifically detect immune cells expressing mIgE while excluding those that capture secreted IgE, such as mast cells, basophils, and eosinophils [120]. FcɛRIα-based CAR-T cells that also express co-stimulatory domains 4-1BB and/or CD28 present a promising avenue for developing adoptive T-cell therapy for allergic diseases [119].

Type 2 innate lymphoid cells (ILC2s) play crucial roles in the development of the Th2 immune response, producing cytokines in a non-allergen-specific manner [106]. ILC2s are activated by allergen-induced, epithelial-derived cytokines such as IL-33 and thymic stromal lymphopoietin, along with interactions with lymphocytes and dendritic cells [121]. Compared to T and B cell-mediated allergic responses, ILC2-mediated responses are rapid and independent of antigen stimulation. Reversing the blockade of certain immune checkpoint molecules on ILC2s is being investigated for its potential therapeutic efficacy against allergic diseases. Recent strategies, including cross-linking immune checkpoint molecules [121] or using agonists of checkpoint molecules like PD-1 to activate inhibitory pathways [106, 122], have shown promise in preclinical studies to suppress allergic inflammation. Further investigations are needed to assess their safety and efficacy in humans.

## Overcoming immunotherapy roadblocks through AI-driven personalized approaches

Immunotherapy does not yield uniform efficacy across all patients [123, 124]. A common complication associated with immunotherapy is the development of irAEs [125]. These events result from enhanced activation of the immune system, primarily manifesting as inflammatory responses. While irAEs are typically managed with steroids, they can occasionally take on a more aggressive form [17]. As interest in advanced immunotherapeutic approaches increases, there is a pressing need to identify patients who will benefit most from immunotherapy while minimizing the risk of untreatable irAEs, thereby avoiding unnecessary healthcare costs [1].

The therapeutic efficacy of immunotherapy is influenced by a complex interplay of factors, collectively referred to as a patient’s immune landscape [126, 127, 128, 129]. Our understanding of these factors is still in its nascent stages. Techniques such as epigenetic profiling, proteomics, single-cell transcriptomics, TCR repertoire analysis, and high-dimensional imaging of immune cells are being employed to gain insights into this intricate immune landscape [17]. Identifying specific signatures or biomarkers can assist clinicians in anticipating immunotherapy outcomes.

In cancer, the immune landscape is primarily shaped by the expression of checkpoint molecules, tumor immunogenicity (including mutational burden and antigen presentation), and the tumor microenvironment [126, 130, 131, 132]. Moreover, genetic factors, epigenetic modifications, and the gut microbiome have been shown to influence immunotherapy outcomes, particularly in the context of ICI therapy [130].

Research focused on identifying and validating potential biomarkers to predict sensitivity to cancer immunotherapy has predominantly centered on the application of ICI therapy. Currently, the FDA has approved three biomarkers—microsatellite instability, PD-L1 expression, and tumor mutational burden (TMB)—for predicting responses to ICI therapy [133]. However, none of these biomarkers consistently correlate with treatment efficacy across diverse clinical settings, primarily due to factors such as tumor heterogeneity and temporal variability [134]. By contrast, research on predictive biomarkers for irAEs is still in its early stages, with no biomarkers currently recognized or validated for clinical use [135].

Advancements have also been made in identifying predictive biomarkers for autoimmune diseases such as RA, MS and allergies. Nonetheless, there are currently no biomarkers approved for routine clinical use [136, 137, 138]. Nevertheless, several promising biomarker candidates have emerged (see Table 3).

**Table 3:** Candidate biomarkers for predicting immunotherapy outcomes in autoimmune diseases

**S. No.**	**Disease**	**Candidate biomarker**	**Biomarker type**	**Biomarker relevance**	**Clinical implementation**	**Reference**
1	RA	Anti-CCP and RF	Protein	Diagnostic and prognostic	Clinically utilized diagnostic biomarker; Investigational prognostic biomarker (tested in small/moderate sized cohorts)	[139, 140, 141, 142]
2	RA	HLA-DRB1*01, HLA-DRB1*04, HLA-DRB1*10 and HLA-DRB1*14:02	Genetic	Prognostic	Investigational (tested in small cohorts)	[142, 143, 144]
3	MS	NFL	Protein	Prognostic	Investigational (tested in large and small cohorts)	[145, 146, 147, 148]
4	MS	GFAP	Protein	Prognostic	Investigational (tested in small cohorts)	[149]
5	MS	CXCL13	Protein (Chemokine)	Predictive and prognostic	Investigational (tested in small cohorts)	[200]
6	MS	CHI3L1	Protein	Prognostic	Investigational (tested in small cohorts)	[150]
7	Asthma	IgE	Humoral	Diagnostic, prognostic and predictive	Clinically utilized diagnostic biomarker; Investigational prognostic biomarker (tested in small/medium cohorts)	[138, 151]
8	Asthma	Eosinophil count	Cellular	Diagnostic, prognostic and predictive	Clinically utilized diagnostic biomarker; Investigational prognostic biomarker (tested in small and large cohorts)	[152, 153, 154, 155]
9	Asthma	FeNO	Metabolic	Diagnostic, prognostic and predictive	Clinically utilized diagnostic biomarker; Investigational prognostic and predictive biomarker (tested in large cohort)	[156, 157, 155]

Abbreviations: RA: Rheumatoid arthritis; Anti-CCP: Anti-cyclic citrullinated peptide; RF: Rheumatoid factor; HLA-DRB1: Human leukocyte antigen DR beta 1; MS: Multiple sclerosis; NFL: Neurofilament light chain; GFAP: Glial fibrillary acidic protein; CXCL13: C-X-C motif chemokine ligand 13; CHI3L1: Chitinase-3-like protein 1; IgE: Immunoglobulin E; FeNO: Fractional exhaled nitric oxide.

It is increasingly evident that a single biomarker is unlikely to provide an accurate estimate of response to immunotherapy [130]. Tailored treatments that consider a patient’s unique characteristics and immune status can enhance outcomes and mitigate unnecessary risks. Innovations in genomic sequencing and immune profiling techniques have facilitated personalized approaches to immunotherapy, aiming to predict patient responses to therapies like checkpoint inhibitors by identifying neo-antigens and developing novel antibodies [158, 159]. Successful integration of sequencing data and AI in predicting immunotherapy outcomes in cancer patients has been demonstrated in various studies.

Analyzing somatic mutations, including base substitutions, rearrangements, insertions, and deletions (indels), in conjunction with AI techniques has shown promise in predicting outcomes associated with PD-1 ICI therapies [160, 161]. Similarly, transcriptomics or RNA sequencing data combined with machine learning (ML) has enabled the differentiation of responders from non-responders while elucidating the mechanisms by which tumors develop resistance to immunotherapy across diverse cancer types [162, 163]. A deep learning model developed using specific TCR repertoire sequences and Human Leukocyte Antigen (HLA) typing has facilitated patient stratification and prediction of responses to ICI therapy in melanoma patients [164].

Currently, multi-omics profiling of tumors and/or the tumor microenvironment (TME) is garnering significant attention for the identification of novel biomarkers in cancer immunotherapy [124, 134]. The complexity of the data obtained makes it challenging for human experts to capture intricate signals across these datasets; however, AI excels in processing such information [124]. Employing a multi-modular approach, with AI models built and trained on multi-omics datasets, can yield a more comprehensive, accurate, and clinically useful framework for prognostic modeling in immunotherapy [159].

In addition to oncology, there is significant interest in the development of predictive models for immunotherapy outcomes related to allergies [172, 174, 182] and rheumatoid arthritis [169, 183]. Similar methodologies are being adapted to forecast immunotherapy outcomes for autoimmune diseases, including T1DM and MS [184-188].

Table 4 summarizes research studies focused on developing predictive AI models to evaluate the success of immunotherapy across various diseases. As evidenced, omic datasets are extensively utilized in model construction, either independently or in combination with other biological information. Prognostic AI models built on routine clinical data, which is relatively accessible and cost-effective, have been shown to perform comparably to multi-omics-based AI models. However, reliance on sequencing information is perceived as necessary for providing an in-depth explanation of predictions made [165]. Moreover, models developed using a multi-modular approach tend to exhibit greater reliability. Therefore, for effective predictions and broader adoption of predictive AI models, the integration of omics and real-world clinical data in model construction is essential. Presently, ICI therapy remains at the forefront of most multi-omics ML models developed for predicting immunotherapy outcomes in cancer. Recently, there has also been growing interest in leveraging ML and multi-omics to interpret the TME in the context of CAR-T cell therapy for solid tumors [181].

**Table 4:** Representative prognostic AI models for predicting immunotherapy outcomes in cancer and autoimmune diseases, along with their key features

**Input dataset**	**Outcome**	**AI model**	**Validation**	**Performance metric**	**Model interpretability**	**Disease**				
						**Cancer**	**Allergy**	**RA**	**T1DM**	**MS**
Omics	Distinguishes responders from non-responders for anti-PD-1 therapy against skin cancer	10 different models; multilayer perceptron neural network and AdaBoost were observed to be most accurate	Internal	96.7% testing accuracy for basal cell carcinoma and 60.7% testing accuracy for melanoma	Moderate	[162]				
	Predicts success of anti PD/PD-L1 therapy in NSCLC patients	Convolutional neural network (CNN), logistic regression, support vector machine (SVM), and random forest (RF) models	External cohort	Area under the curve (AUC) in the range of 0.959-0.965 for the different models	Moderate	[160]				
	Predicts success of anti PD/PD-L1 therapy	9 different models; Least Absolute Shrinkage and Selection Operator (LASSO) offered the highest prediction performance	Internal	AUC of 0.93	High	[161]				
	Distinguishes responders from non-responders for ICI therapy	SVM, XGBoost and deep neural networking	External cohort (leave-one-out cross-validation)	Accuracy of 100%	Moderate	[163]				
	Enables patient stratification and predicts success of ICI therapy	CNN, variational autoencoders, and multi-instance learning algorithm	External cohort	AUC of 0.86	Moderate	[164]				
Multi-omics	Enables a consistently effective therapeutic strategy	REFLECT: combination of sparse hierarchical clustering and LASSO algorithm	*In vitro* cell lines, patient-derived xenografts and clinical trial data	Average concordance of 83%	High	[166]				
	Predicts the efficacy of different anti-cancer therapeutic regimens	StepCox (forward) + Ridge algorithm	Multiple external datasets and previously published models	AUC >0.65	High	[167]				
	Predicts success of anti-PD-1 therapy	LASSO regression	Internal	AUC in the range of 0.62--0.64 for the different models	High	[168]				
	Predicts response to Adalimumab and Etanercept in RA patients	RF-ML Model	Internal	Models displayed accuracy in the range of 72--88%	Moderate			[169]		
Immune cell profiling	Enables stratification of T1D patients into responders/non-responders w.r.t Abatacept treatment	Gradient boosting model	Internal and external	AUC of 0.81	Moderate				[170]	
						**Cancer**	**Allergy**	**RA**	**T1DM**	**MS**
Clinical Parameters	Predicts outcome of subcutaneous immunotherapy in paediatric allergic rhinitis patients	Binary improved sine cosine algorithm (birSCA)- SVM algorithm	Internal	Accuracy of 88.99%	Moderate		[171]			
	Predicts the efficacy of mite subcutaneous immunotherapy in asthma	Disperse Foraging Strategy Salp Swarm Algorithm-Kernel Extreme Learning Machine (DFSSSA-KELM)	Internal	Accuracy of 87.18%	Moderate		[172]			
	Predicts a disease activity score for RA patients undergoing immunotherapy	8 regression models; ridge regression model was observed to be most accurate	Internal and external	Mean absolute error values for the different models ranged between 0.633--0.857	High			[173]		
Immunoassays	Predicts sustained unresponsiveness to milk oral immunotherapy	Elastic net algorithm (logistic regression method)	Internal	Average accuracy of 92%--95%	High		[174]			
Multi-Dimensional	Provides a risk score for response to immunotherapeutic agents	Random Survival Forest (RSF) algorithm	Internal	Not listed	High	[175]				
	Predicting overall survival and success of ICI therapy	Deep learning-natural language processing and RSF	Internal and external	AUC > 0.95	Low	[176]				
	Differentiates responders from non-responders and enables patient stratification into high and low-risk groups for anti-PD-1/PD-L1 therapy	SimTA: deep learning model with temporal attention module assembled using multilayer perceptron	Internal	SimTA60d- AUC of 0.77 and SimTA90- AUC of 0.80	Low	[177]				
	Predicts patient’s survival post ICI-therapy	SCORPIO: ensemble of ridge logistic regression, SVM and RF with soft voting algorithm	Internal and external- test sets and cohorts	Internal: median pan-cancer AUCs of 0.759 and 0.641 for overall survival and clinical benefit respectively. External test set: median pan-cancer AUC of 0.725 for overall survival	High	[165]				
	Predicts response to SCIT	RF modelling-ML algorithm	Internal and external cohort	Internal: AUC of 0.899 and external: AUC of 0.893	Moderate		[178]			
	Predicts clinical response to Adalimumab and Etanercept therapy in RA patients	RF algorithm	Internal	Accuracy of the different models ranged between 79%--88%	Moderate			[169]		
	Predicts response to Fingolimod therapy in MS patients	RF algorithm	External test set	AUC of 0.71	Moderate					[179]
	Predicts response to TNF inhibitor therapy in RA patients	Linear regression, RF, XGBoost and CatBoost	External cohort	AUC values of 0.7 and 0.71	Moderate			[180]		

Abbreviations: AI: Artificial intelligence; anti-PD-1: Anti–programmed cell death protein 1; PD-L1: Programmed death-ligand 1; NSCLC: Non-small cell lung cancer; CNN: Convolutional neural network; SVM: Support vector machine; RF: Random forest; RF-ML: Random forest–based machine learning; ML: Machine learning; AUC: Area under the curve; ICI: Immune checkpoint inhibitor; RA: Rheumatoid arthritis; T1D: Type 1 diabetes; T1DM: Type 1 diabetes mellitus; MS: Multiple sclerosis; LASSO: Least absolute shrinkage and selection operator; XGBoost: Extreme gradient boosting; CatBoost: Categorical boosting algorithm; REFLECT: Name of an AI framework combining sparse hierarchical clustering and LASSO; StepCox: Stepwise Cox proportional hazards regression: W;r.t.: With respect to; birSCA: Binary improved sine cosine algorithm; DFSSSA: Disperse Foraging Strategy Salp Swarm Algorithm; KELM: Kernel Extreme Learning Machine; DFSSSA-KELM: Disperse Foraging Strategy Salp Swarm Algorithm–Kernel Extreme Learning Machine; RSF: Random survival forest; DL: Deep learning; NLP: Natural language processing; SimTA: Deep learning model with temporal attention module; SCIT: Subcutaneous immunotherapy; TNF: Tumor necrosis factor; SCORPIO: Ensemble prognostic model (ridge logistic regression, SVM and RF with soft voting).

Based on their explainability, AI-ML algorithms can be broadly categorized into transparent and opaque categories. Transparent algorithms provide end-to-end interpretability but may lack the proficiency of opaque algorithms in handling complex tasks with higher accuracy. Examples of transparent algorithms include K-Nearest Neighbors, Naive-Bayes, Logistic Regression (LR), and Decision Trees, while opaque algorithms include Random Forests (RFs), Support Vector Machines (SVMs), and Deep Learning (DL) methods such as Convolutional Neural Networks (CNNs) and Multilayer Perceptrons (MLP) [201].

Wider user adoption of AI-ML prognostic tools necessitates the establishment of clinician trust, which requires thorough validation and clear communication of AI decision systems. Achieving superior predictive capability entails the simultaneous assessment of multiple biological parameters, necessitating the use of complex or ensemble ML methods. This complexity can render the tools less transparent and obscure their decision-making processes. Therefore, striking a balance between explainability and the accuracy of AI systems is essential [201].

While clinicians do not need to grasp the entire algorithm, they must understand how biological factors influence the decision-making process. Several methods have been developed to enhance the interpretability of ML models, which are briefly discussed here. One approach involves post-hoc analysis, where insights are derived without a precise focus on the internal workings of the model. Most of these methods are model-agnostic, meaning they can be applied to various models without requiring direct access to the internal model structure. Conversely, model-specific methods cater to particular ML algorithms and yield more precise interpretations. Additionally, ML explanation methods can be categorized based on whether they provide explanations for individual samples (local) or for the overall functioning of the model (global interpretation) [202]. Shapley Additive Explanations (SHAP) and Local Interpretable Model-Agnostic Explanations (LIME) are widely used interpretability methods in the healthcare-AI sector, and they have been applied to develop explainable AI models for predicting immunotherapy outcomes [165, 168, 169, 203, 204].

## Challenges and opportunities

Advances in immunotherapy, coupled with the increasing accessibility of AI and ML algorithms, are reshaping treatment paradigms for various diseases and facilitating precision medicine. However, the integration of these fields is still in its infancy, facing challenges such as data privacy and security, algorithmic bias, and integration into clinical workflows.

### Data quality and quantity

Data bias can hinder the development of generalized AI models. Constructing robust ML models requires large cohort sizes and comprehensive patient profiling, which necessitates substantial financial investments and collaborative efforts [189]. Currently, many AI models exhibit limited accuracy due to the lack of extensive, validated datasets from large clinical trials [189, 159]. Recent studies aim to address these challenges by utilizing training data from multiple centers and deploying transfer learning algorithms. Nonetheless, biases in funding, resource allocation, and ethnic disparities may persist [190, 191, 192].

### Model interpretability

The “black-box nature” of AI models, where internal analytical processes remain opaque, presents a significant barrier to their broader adoption [159]. This is particularly critical in healthcare, where decision-making carries substantial risks, and patient consent is influenced by understanding the underlying mechanisms [193]. As noted, responses to immunotherapy rely on numerous interconnected and variable parameters, rendering the data non-linear and complex. This multidimensional information is interpreted using ML and DL algorithms, such as CNNs and the intricacies involved are not always comprehensible, especially to non-experts. Furthermore, delivering understandable explanations of model workflows without compromising accuracy poses a challenge [194]. A clear understanding of the decision-making process is likely to promote the wider adoption of predictive models in clinical practice [192]. Implementing explainable AI (XAI) approaches, where each step of the ML process is traceable with explanations, may provide a promising solution [159]. However, the debate surrounding explainable AI as a remedy for the “black-box problem” remains unresolved, primarily due to the diverse concerns this issue raises for various stakeholders, including developers, clinicians, patients, and regulatory authorities [193, 194].

### Regulatory framework and ethical aspects

Currently, regulations governing the use of AI in healthcare are in a nascent stage. Presently, AI applications in healthcare are primarily regulated under the software as a medical device (SaMD) criteria established by the International Medical Device Regulators Forum (IMDRF) and the FDA [195]. Recently, principles for good machine learning practice (GMLP) have also been introduced by IMDRF, serving as a foundation for advancing GMLP standards through collaboration among various international standards and regulatory organizations.

Additionally, the newly enacted European Union (EU) AI Act represents the world’s first comprehensive legal framework for AI. The EU AI Act is expected to drive changes in the AI-based healthcare technology sector globally, as it establishes benchmarks for the development and utilization of AI. This act is particularly significant for the healthcare sector, as it specifically addresses medical AI technologies, unlike existing regulations [196]. According to the provisions of this act, prognostic AI models, such as those predicting immunotherapy outcomes, are classified as “high risk.” This classification obligates developers to present model interpretability reports and longitudinal safety data to assess clinical feasibility [196, 197].

AI tools face serious accountability issues due to their “black-box nature” and their reliance on biased inputs, which can lead to biased outputs [198]. In instances where AI assistance results in unintentional harm to patients, liability should not rest solely with clinicians but also with the manufacturers of the AI tools, the clinicians’ organizations, and the healthcare system as a whole. To address the unforeseen challenges posed by the application of AI in healthcare, it is crucial to reevaluate existing healthcare ethical guidelines [199].

Another significant ethical concern pertains to data confidentiality and security. AI tools are trained on extensive, sensitive patient information; unintended and unauthorized access to this data could favor certain stakeholders and compromise patient interests. It is imperative to safeguard patient interests by implementing robust security measures and adhering to regulatory laws [192].

## Conclusion

While immunotherapy is often associated with cancer treatment, it is increasingly being explored for various other diseases, including asthma, MS, arthritis, HIV and tuberculosis. Although the overall results from these explorations have been promising, setbacks encountered in the clinical application of novel immunotherapeutics for cancer must be acknowledged, alongside the associated high costs. To ensure that immunotherapy is safe, effective, and ultimately successful across a broad spectrum of diseases, a personalized approach is essential. Personalized immunotherapy is supported by advances in omic profiling, biomarker identification, and the development of prognostic models, with the latter two enabled by AI. Concerningly, the pace of advancements in AI does not match the establishment of regulatory frameworks. This discrepancy, along with challenges related to interpretability, data quality (bias), and ethics, presents formidable barriers to the application of AI in healthcare. Overcoming these obstacles will require collaborative efforts from clinicians, research scientists, developers, regulatory agencies, and policymakers.

In summary, this article provides a comprehensive overview of how immunotherapy is being utilized or investigated for treating conditions beyond cancer. It further explores how AI can facilitate the broader implementation of immunotherapy and the challenges associated with this integration. However, the article is limited by its narrative nature and the absence of a standardized methodology or quality appraisal process in its framework.

## Data Availability

No datasets were generated or analyzed during the current study.
